# The economic burden of Myasthenia gravis from the patient´s perspective and reflected in German claims data

**DOI:** 10.1038/s41598-025-91372-7

**Published:** 2025-02-25

**Authors:** Sophie Lehnerer, Meret Herdick, Antje Mevius, Ulrike Grittner, Pauline Gansel, Lars Joeres, Jutta Biskup, Andreas Meisel

**Affiliations:** 1 Department of Neurology with Experimental Neurology, Charité – Universitätsmedizin Berlin, Corporate Member of Freie Universität Berlin and Humboldt-Universität zu Berlin, Charitéplatz 1, 10117 Berlin, Germany; 2 Neuroscience Clinical Research Center, Charité – Universitätsmedizin Berlin, Corporate Member of Freie Universität Berlin and Humboldt-Universität zu Berlin, Charitéplatz 1, 10117 Berlin, Germany; 3 Center for Stroke Research Berlin, Charité – Universitätsmedizin Berlin, Corporate Member of Freie Universität Berlin and Humboldt-Universität zu Berlin, Charitéplatz 1, 10117 Berlin, Germany; 4 Digital Health Center, Berlin Institute of Health at Charité – Universitätsmedizin Berlin, Charitéplatz 1, 10117 Berlin, Germany; 5 IPAM e.V., Alter Holzhafen 19, 23966 Wismar, Germany; 6 Institute of Biometry and Clinical Epidemiology, Charité – Universitätsmedizin Berlin, Corporate Member of Freie Universität Berlin and Humboldt-Universität zu Berlin, Charitéplatz 1, 10117 Berlin, Germany; 7 Charité – Universitätsmedizin Berlin, Corporate Member of Freie Universität Berlin and Humboldt-Universität zu Berlin, 10117 Berlin, Germany; 8 UCB Pharma, Rolf-Schwarz-Schütte-Platz 1, 40789 Monheim am Rhein, Germany

**Keywords:** Myasthenia gravis, Burden of disease, Economic burden, Treatment costs, Neuromuscular disease, Autoimmunity

## Abstract

Myasthenia gravis (MG) is a neuromuscular disorder resulting in exertion-dependent muscle fatigability. Affecting all age groups MG is a chronic disease with unpredictable fluctuations and a range of symptom expression which can require costly immunosuppressive therapy and intensive care critically influencing economic burden. Here we aim to elucidate both the personal and societal economic burden of MG. Data were used from two sources: (1) a cross sectional study of MG patients registered in the German Myasthenia Society (DMG) collected by a survey in 2019 (n = 1660), and (2) from a dataset provided by a German health insurance company covering the period from 01/01/2014 to 31/12/2019 (n = 750) and categorized to similar subgroups for comparison. Individual and societal economic burden was highest in patients with intensified medical treatment. Absence days from work per patient year in the first year after index in claims data were 67.1 days (95% CI 66.1–68.2) (referring to n = 260 non-retired patients). Of 686 patients with early onset (< 50 years) MG 260 (41.2%) reported loss of income due to MG in DMG data. Average inpatient costs from claims data in patients with intensified treatment (38,669€; 95% CI 38,627–38,712€) were particularly high compared with standard treatment (2980€; 95% CI 2975–2986€) and to no MG-related treatment (887€; 95% CI 877–886€). MG represents a major economic burden for the individual and the health care system. The costs are positively associated with the severity of disease. The economic burden is particularly high for working patients. UCB Pharma provided financial support for the subanalysis, but was not involved in the design of the study.

*Trial registration*: clinicaltrials.gov, NCT03979521, submitted: June 7, 2019, first patient enrolled: May 1, 2019, https://clinicaltrials.gov/ct2/show/NCT03979521.

**Supplementary Information:**

The online version contains supplementary material available at 10.1038/s41598-025-91372-7.

## Introduction

Myasthenia gravis (MG) is a rare, autoimmune neuromuscular disease characterized by dysfunction and damage at the neuromuscular junction leading to exertion-dependent muscle fatigability^1^. Depending on the affected muscle groups patients are characterized as having ocular MG (oMG) or generalized MG (gMG). Life threatening exacerbation and crisis are a particular risk whenever respiratory or bulbar muscles are involved^2^. MG can manifest at a young age and, due to its chronic nature, often requires lifelong treatment. The management of MG aims to minimize symptom expression with a minimum of side effects, but this is often not achieved with the current standard of care, including acetylcholinesterase inhibitors, immunosuppressants, biologics, intravenous immunoglobulins and plasma exchange^3^. The financial aspect of managing MG is becoming increasingly relevant with emerging novel therapies both adding value to standard of care and costs to the healthcare system^4^. The precise treatment costs associated with MG remain inadequately understood, compounded by significant national disparities and a lack of representative data in Germany. This knowledge gap not only burdens the healthcare system but also contributes to individual economic burdens, particularly given that patients often become affected during their prime working years, and the severity and unpredictability of symptomatology vary greatly. So far we know that inpatient costs in MG have been reported to exceed outpatient and rehabilitation costs^5^. Disease severity and assistance in activities of daily living were identified as potential driving factors of MG treatment costs in Germany^5^. Beyond those costs resulting from systemic expenditures e.g. health insurance costs, inability to work results both in societal as well as individual^6^. More detailed exploration of personal costs in MG has not been performed.

The main objective of this study is to characterise the economic burden of the MG population from a patient and societal perspective with two corresponding datasets, elucidating upon costs at a point in time when targeted treatment options beyond rituximab in MG were limited to eculizumab. To scrutinise differences in MG subgroups regarding their economic burden under the conditions of the German healthcare system, this study analyses in secondary objectives subgroups of patients with oMG and gMG, patients with early onset and late onset MG (EOMG, LOMG), and patients with standard treatment, intensified treatment or no MG-related treatment. More specifically, we aim to characterize the economic burden (costs for health and medical aids, for privately borne additional expenses such as travel costs, home renovation, childcare costs), fear of poverty in old age, care dependency levels, sickness-related absences and influence on professional status in relation to MG and its subgroups by analyzing two datasets, one obtained from a cross-sectional study of MG patients registered with the German Myasthenia Society and the other from a German health insurance company.

## Methods

Data were used from two sources: (1) a cross sectional study of MG patients registered in the German Myasthenia Society (DMG) collected by a survey in 2019^7^, and (2) from a dataset provided by a German health insurance company (AOK PLUS^8^) covering the time window from 01/01/2014 to 31/12/2019.

### DMG dataset

#### Standard protocol approvals, registrations, and patient consent

The study was approved by the ethics committee at Charité – Universitätsmedizin Berlin (no. EA1/008/19). No written informed consent was obtained from the study participants since the data collection was completely anonymous. The study was conducted in accordance with the declaration of Helsinki and registered on clinicaltrials.gov (NCT03979521). The STROBE reporting guidelines for observational studies have been applied^9^.

#### Study design and setting

This is a sub-analysis of a cross sectional questionnaire-based study that was sent out in May 2019 to 3262 members of the DMG^7^. The study participants received the study information with the questionnaire as well as a pre-stamped envelope addressed to the coordinating study centre. Participants were instructed to return their completed questionnaire without any further identifying information to ensure the anonymity of the survey. No refund was given. Returned questionnaires were accepted within the cut-off date of 31th July 2019.

#### Data sources

The questionnaire contained items on several sociodemographic and disease related dimensions, described in detail in our former publication^7^. For this sub-analysis relevant data was: gender, age, disease duration, disease severity (self-rated in the categories “mild”, “medium”, “severe”), medical treatment, clinical subtype, as well as questions regarding employment and income, health care resource utilization (HCRU), type of transport to treating physician, negotiations with health insurance companies, care dependency level, and ownership of a severely disabled person’s pass. In Germany, people with a chronic illness that limits their participation in the community with a degree of disability of 50 or more can obtain a severely disabled person’s pass, which is a prerequisite for claiming compensation for disadvantages. A person’s need for care is assessed by the medical services of the health insurance funds in Germany. The care dependency level is determined, with care dependency level one having the least restrictions and care dependency level five the highest. Based on this, care benefits are paid by the health insurance companies. All of the questions used for this sub-analysis allowed for checkbox responses, which were always specified as single or multiple choice. The questionnaires were scanned and processed with the software TeleForm (OpenText), version 10.9.1^10^.

#### Standardized scores

To further assess the burden of disease, standardized scores were integrated in the questionnaire, two of which were used in this sub-analysis: MG-QoL15 (Myasthenia gravis quality of life, i.e. MG specific HRQoL)^11^ and MG-ADL (Myasthenia gravis activities of daily living profile)^12^. In the MG-QoL15 (0–60-point scale) and the MG-ADL (0–24-point scale) a high score indicates a worse situation.

#### Statistical analysis

The statistical calculations were performed using IBM SPSS Version 25.0^13^ and R (version 3.5.3)^14^. Depending on the scale and distribution of the outcome variables, appropriate descriptive statistics (mean, standard deviation, median, interquartile range, absolute and relative frequencies) are presented. Missing data are listed in the table as “Unknown” and are not included in the calculation of relative frequencies. We calculated the standardized mean difference (SMD) as standardized effects size measure for quantifying subgroup differences. The SMD is Cohen’s d in the case of comparing two groups in a continuous measure. We used the calculation of the SMD as implemented in the R package *tableone* with extensions of the SMD for nominal data^15^.

#### Subgroup definitions

Patients were queried regarding their specific subtype of MG: ocular (only eyes are affected, oMG) or generalised MG (extremities and/or swallowing/respiratory muscles are affected, gMG). Furthermore, early onset (EOMG) was defined if symptom onset was below the age of 50 years, late onset (LOMG) if patients had their first symptoms at the age of 50 years or later. Another stratification was made based on medical treatment. Patients were categorised as getting standard treatment if they got at least one of the following: pyridostigmine, mycophenolate mofetil, steroids, azathioprine, methotrexate, or ciclosporin A. Patients were categorised as getting intensified treatment if they had received rituximab, eculizumab, intravenous immunoglobulins, plasmapheresis, or immunoadsorption within the last 12 months.

### Claims dataset

#### Data source and study design

This retrospective observational longitudinal study was based on anonymized claims data covering the period from January 1, 2015, to December 31, 2019, provided by the German statutory health insurance fund AOK PLUS. The dataset covers approximately 3.4 million individuals from the German federal states of Saxony and Thuringia, corresponding to around 4% of the total German population and 4.7% of all people with statutory health insurance in Germany. The AOK Plus data was used in previous studies^8^.

In Germany, the majority of the population is covered by statutory health insurance, which provides comprehensive healthcare services. About 10–15% have private health insurance, usually people with higher-income or who are self-employed. Private health insurance often offers broader benefits and higher reimbursement rates, which may affect treatment decisions and access to care. German claims data provide information on patients’ demographics (age, gender, date of death; cause of death is not available) and detailed reimbursement claims on outpatient care, inpatient care, pharmaceuticals, and therapeutic devices. The outpatient care data comprise information on diagnostic and therapeutic procedures, the diagnosis made by an outpatient physician as well as the type of treating physician. The inpatient care data covers information on the date of admission and length of stay, diagnostic and therapeutic procedures. Inpatient and outpatient diagnoses are coded according to the German Modification of the International Classification of Diseases 10th Revision (ICD-10-GM). Data on outpatient prescriptions of reimbursed drugs include information on the date of prescription, the specialty of prescribing physician, and the pharmaceutical reference number (PZN) of the prescribed agents.

#### Study population

Index is defined as date of first diagnosis observed in the database. The study population of incident MG patients included all patients who had received at least one inpatient and/or two confirmed outpatient diagnoses of MG (outpatient diagnoses in two different quarters; ICD-10 G70.0) in the period between 01/01/2015 and 31/12/2019, had a 12-month period without any MG diagnoses prior to the index date, and were continuously insured by the sickness fund 12 months before index date.

#### Subgroup definitions

We aimed to homogenize subgroups between DMG and claims dataset, accordingly we defined subgroups as follows: incident MG patients were stratified by age at time of the diagnosis: early onset MG (EOMG; < 50 years at index date); and late onset MG (LOMG; ≥ 50 years at index date). Another stratification of the study population was made by MG treatment during the first year following the first MG diagnosis: (1) patients without any MG treatment, (2) patients with symptomatic and immunosuppressive standard treatment (pyridostigmine, corticosteroids, azathioprine, mycophenolat mofetil, methothrexate), and (3) patients on intensified treatment (eculizumab, rituximab, intravenous immunoglobulines, plasma exchange, immunoadsorption).

#### Variables and statistical analysis

Incident MG patients were observed starting from their first documented MG diagnosis (index date between 01/01/2015 and 31/12/2019) until 31/12/2019 or end of insurance or death (whichever came first).

Patient characteristics were assessed at the index date. The number of incident patients who received at least one prescription/administration/procedure of the defined MG treatments (identified via ATC or OPS code; Supplement 1) in the 12 months after index date was assessed (stratified by EOMG and LOMG/by MG treatment). For all categorical variables, the absolute and relative frequency of patients in each category were assessed.

For health care resource utilization (HCRU), the number of patients with at least one MG-related outpatient visit (any, including neurologists and GPs) and the related number of visits per patient-year as well as the number of patients with at least one MG-related inpatient stay/intensive care unit (ICU)-stay/emergency room (ER) visit and the respective number of visits per patient-year and the number of days in the hospital per patient-year were assessed. Additionally, MG-related absence from work in the first year after the incident diagnosis was analyzed for all non-retired persons: number of affected patients, number of absences and number of absent days per patient-year. “MG-related” in this context was defined as any utilization (outpatient visits, hospitalizations, absences from work) that was associated with an MG-diagnosis (ICD-10 G70.0).

The cost analyses included expenses for MG-related hospitalizations, outpatient prescriptions, as well as medical aids (e.g., walking aids) and therapeutic remedies (e.g., speech or occupational therapy) within the first year after index date and calculated per patient-year.

## Results

### Patient characteristics in DMG dataset

Based on the inclusion criteria, a total of 1660 patients were eligible for analysis. Mean age of patients was 65.2 years (SD 14.9 years) with an overall gender ratio (female to male) of 1.28 (56.2% vs. 43.8%), with 0.73 (42.1 vs. 57.9%) for oMG and 1.53 (60.5% vs. 39.5%) for gMG (Table 1). More women (79.4%) had EOMG than men (20.6%), whereas more men (64.0%) had LOMG than women (36.0%). The majority of patients received standard treatment (n = 1,256, 76.3%), whereas 10.3% (n = 169) received intensified treatment and 13.4% (n = 221) received no MG-related treatment. Disease duration was longest for patients receiving no MG-related therapy (mean 19.3 years, SD 12.5) compared with those receiving standard (mean 13.0 years, SD 11.4) or intensified treatment (mean 11.1 years, SD 9.5), though the effect size was small (SMD 0.18). More patients receiving intensified treatment reported a severe course of MG (25.0%) than groups with standard (6.8%) or no (9.0%) MG-related treatment. In contrast, patients with no MG-related treatment were more likely to report mild MG (64.2%) compared to MG with standard (47.5%) or intensified (12.6%) treatment. The association of disease severity and treatment intensity had an SMD of 0.89. The overall mean MG-ADL score was 4.2 (SD 3.5), with higher scores in gMG than oMG as well as in patients with intensified compared to standard treatment (Supplement 2). Patients with EOMG reported higher symptom burden (mean MG-ADL 4.8, SD 3.6) than patients with LOMG (mean MG-ADL 3.9, SD 3.1). Assessment of MG-QoL15 revealed an overall mean score of 17.2 (SD 13.4) (Supplement 3). At the time of study participation mean age of patients with LOMG (n = 851, 55.4%; 73.7 years, SD 8.5 years) was higher than in patients with EOMG (n = 686, 44.6%; 54.1 years, SD 13.8 years). Mean disease duration was longer in EOMG patients (19.6 years, SD 13.2 years) than LOMG patients (8.9 years, SD 7.2 years).

**Table 1:** Patient characteristics of DMG dataset (age, gender, disease duration, severity of Myasthenia gravis) in overall study population and different subgroups.

	Overall	oMG	gMG	SMD^1^	No MG-related treatment	Standard treatment	Intensified treatment	SMD^2^	EOMG	LOMG	SMD^3^
n	1660	388	1272	0.31	221	1256	169		686	851	− 1.7
Age in years, mean (SD)	65.2 (14.9)	68.5 (12.3)	64.1 (15.4)		65.2 (15.0)	57.6 (16.1)	54.1 (13.8)	0.56	54.1 (13.8)	73.7 (8.5)	
Unknown	11	2	9		1	1	4		7	11	
Gender											
Female	931 (56.2)	163 (42.1)	768 (60.5)	− 0.37	138 (62.4)	669 (53.4)	116 (68.6)	0.32	545 (79.4)	305 (36.0)	0.98
Male	725 (43.8)	224 (57.9)	501 (39.5)		83 (37.6)	583 (46.6)	53 (31.4)		141 (20.6)	542 (64.0)	
Diverse	0 (0.0)	0 (0.0)	0 (0.0)		0 (0.0)	0 (0.0)	0 (0.0)		0 (0.0)	0 (0.0)	
Unknown	4	1	3		0	4	0		0	4	
Disease duration in years, mean (SD)	13.6 (11.6)	12.1 (9.7)	14.0 (12.1)	− 0.18	19.3 (12.5)	13.0 (11.4)	11.1 (9.5)	0.18	19.6 (13.2)	8.9 (7.2)	1.0
Unknown	46	8	38		13	30	3		11	14	
Severity of Myasthenia gravis, n (%)				− 0.48				0.89			0.11
Mild	733 (45.6)	238 (63.1)	495 (40.3)		129 (64.2)	582 (47.5)	21 (12.6)		293 (43.8)	404 (49.1)	
Medium	728 (45.3)	123 (32.6)	605 (49.2)		54 (26.9)	559 (45.7)	105 (62.9)		310 (46.3)	347 (42.2)	
Severe	145 (9.0)	16 (4.2)	129 (10.0)		18 (9.0)	83 (6.8)	41 (25.0)		66 (9.9)	71 (8.6)	
Unknown	54	11	43		20	32	2		17	29	

^1^Comparing gMG and oMG.

^2^Comparing standard and intensified treatment.

^3^Comparing EOMG and LOMG.

### Patient characteristics in claims dataset

In the period between 01/01/2015 and 31/12/2019 n = 775 received a first diagnosis of MG (= index). This study population of incident MG patients included all patients who had received at least one inpatient and/or two confirmed outpatient diagnoses of MG (outpatient diagnoses in two different quarters; ICD-10 G70.0). The mean age at index date was 66.9 years (SD 16.6 years) and the gender ratio (female to male) of affected patients (49.4%, 50.6%) was 0.98 (Table 2). Most patients were treated with standard treatment (57.7%) while 29.5% had no MG-related treatment and 12.8% had intensified treatment. Mean age at index date was higher for patients receiving intensified treatment (mean 72.1 years, SD 13.7) than those with standard (mean 66.5 years, SD 16.0) or no MG-related treatment (mean 65.4 years, SD 18.2). Incidence of EOMG (15.5%) was lower than LOMG (84.5%). By definition, age of patients at index date with LOMG (72.4, SD 10.6) was higher than EOMG (36.7, SD 9.2). The gender ratio (female to male) was 1.45 (59.2% vs. 40.8%) in EOMG and 0.91 (47.6% vs. 52.4%) in LOMG.

**Table 2:** Patient characteristics of claims dataset (age, gender, observation days in the first year after index date) in overall study population and different subgroups.

	All incident^1^	No MG-related treatment	Standard treatment	Intensified treatment	SMD^2^	EOMG	LOMG	SMD^3^
n	775	229	447	99		120	655	
Age in years at index date^4^, mean (SD)	66.9 (16.6)	65.4 (18.2)	66.5 (16.0)	72.1 (13.7)	0.38	36.7 (9.2)	72.4 (10.6)	3.60
Gender n (%)								
Female	383 (49.4)	137 (59.8)	204 (45.6)	42 (42.4)	1.08	71 (59.2)	312 (47.6)	0.23
Male	392 (50.6)	92 (40.2)	243 (54.4)	57 (57.6)		49 (40.8)	343 (52.4)	

^1^The cohort of incident MG patients included all patients who had received at least one inpatient and/or two confirmed outpatient diagnoses of MG (outpatient diagnoses in two different quarters; ICD-10 G70.0) in the period between 01/01/2015 and 31/12/2019, had furthermore a 12-months period without any MG diagnoses prior to the index date, and were continuously insured by the sickness fund 12 months before index date.

^2^Comparing standard and intensified treatment.

^3^Comparing EOMG and LOMG.

^4^Index date = first diagnosis reported in claims data.

### Economic burden

#### Work and income

**Fig. 1:**
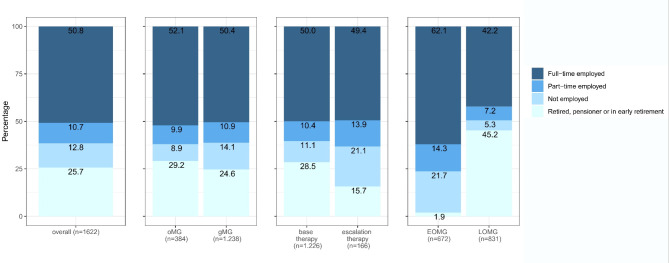
Employment before onset of Myasthenia gravis (DMG dataset) in overall study population and different subgroups.

**Fig. 2:**
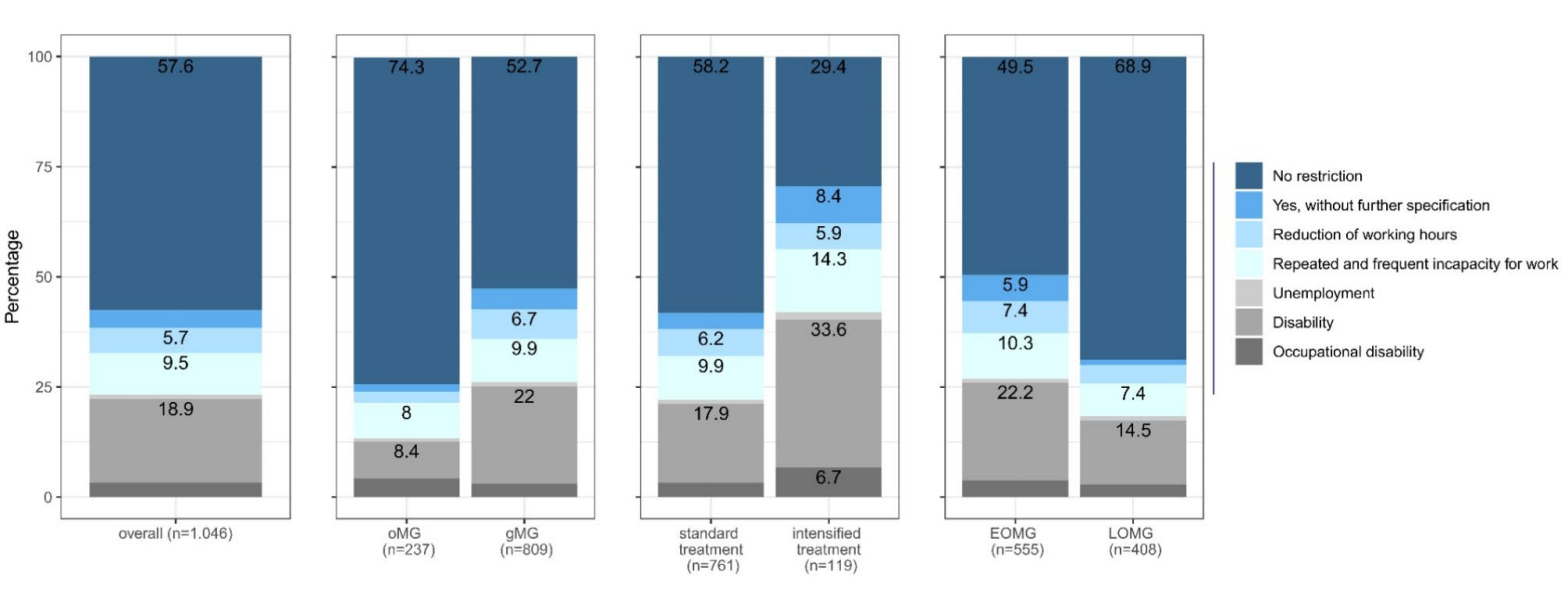
Restrictions on employment due to Myasthenia gravis (DMG dataset) in overall study population and different subgroups.

For the 74.3% of non-retired participants in the DMG cohort (Fig. 1), there was restriction on employment in 42.4% (Fig. 2). MG led to disability in 18.9% of the affected working patients and resulted in repeated and frequent incapacities for work in 9.5% and a reduction of working hours in 5.7% (Fig. 2). Most pronounced differences in employment status before and after onset of MG were found in the comparison of patients with standard treatment where 58.2% of working patients experienced no restrictions on employment. On the other hand only 29.4% of patients in the group with intensified treatment experienced no restriction on their employment due to MG, with the largest proportion reporting disability due to MG (33.6%).

Overall 29.0% of study participants experienced income losses due to MG (Table 3). Income loss was more common in participants with gMG (32.6%) than those with oMG (17.3%), with relatively small effect size (SMD − 0.36), and more common with medium effect sizes with higher therapy efforts (no MG-related treatment 19.8%, standard treatment 26.8%, intensified treatment 53.9%; SMD 0.57), and with EOMG compared to LOMG (EOMG 41.2%, LOMG 18.6%; SMD 0.51). Most participants (87.0%) reported that their monthly income was sufficient, but 25.0% stated that their standard of living did not remain the same as before disease onset (Table 3). Similar to total income loss, the results for maintaining the same standard of living were less favorable for patients with gMG (oMG 83.9%, gMG 71.6%; SMD -0.30), escalated treatment (no MG-related treatment 83.2%, standard treatment 76.4%, intensified treatment 52.3%; SMD 0.52), and EOMG (EOMG 69.3%, LOMG 79.5%; SMD 0.24), the latter difference being of a small effect size compared to a medium effect size in the other comparisons (Table 3). Fear of poverty in old age was more prevalent among participants with EOMG (41.7%) than LOMG (17.7%) with medium effect size of SMD 0.54, of whom 69.4% and 61.8% respectively attributed this fear to MG indicating this effect to be largely independent of EOMG/LOMG-subtype (SMD 0.16).

**Table 3:** Loss of income, self-covered costs and fear of poverty in old age of the DMG dataset in overall study population and different subgroups.

	Overall n = 1,660	oMG n = 388	gMG n = 1,272	SMD^1^	No MG-related treatment n = 221	Standard treatment n = 1,256	Intensified treatment n = 169	SMD^2^	EOMG n = 686	LOMG n = 851	SMD^3^
Loss of income due to MG, *yes* n (%)	440 (29.0)	63 (17.3)	377 (32.6)	− 0.36	39 (19.8)	310 (26.8)	83 (53.9)	0.57	260 (41.2)	146 (18.6)	0.51
Unknown	141	24	117		24	100	15		55	64	
Monthly income sufficient, *yes* n (%)	1,258 (87)	312 (90.7)	946 (86.5)	− 0.13	169 (92.3)	966 (87.9)	115 (79.3)	0.23	502 (83.8)	681 (91.4)	0.23
Unknown	222	44	178		38	157	24		87	106	
Monthly income sufficient for same standard of living as before MG, *yes* n (%)	1,072 (75)	287 (83.9)	785 (71.6)	− 0.30	154 (83.2)	832 (76.4)	79 (52.3)	0.52	411 (69.3)	598 (79.5)	0.24
Unknown	222	46	176		36	167	18		93	99	
Fear of poverty in old age, (*yes* n (%)	483 (30)	90 (23.6)	393 (31.4)	− 0.17	50 (23.3)	355 (28.7)	74 (44.0)	0.32	284 (41.7)	148 (17.7)	0.54
Unknown	27	7	20		6	20	1		5	17	
If yes: MG is reason for this fear, *yes* n (%)	317 (67)	47 (54.0)	270 (69.4)	− 0.32	26 (53.1)	224 (64.2)	64 (86.5)	− 0.54	195 (69.4)	89 (61.8)	0.16
N.a./Unknown	1,184	301	883		172	907	95		405	707	
Amount of self-covered costs due to MG, n (%)				− 0.32				0.58			0.06
No costs	321 (20)	91 (23.9)	230 (18.6)		119 (56.9)	191 (15.5)	11 (6.6)		141 (20.9)	162 (19.5)	
Less than 100 EUR/Month	868 (54)	226 (59.5)	642 (51.8)		60 (28.7)	733 (59.6)	70 (42.2)		362 (53.6)	443 (53.4)	
100–200 EUR/Month	324 (20)	46 (12.1)	278 (22.4)		22 (10.5)	232 (18.9)	64 (38.6)		134 (19.8)	165 (19.9)	
More than 200 EUR/Month	106 (6.5)	17 (4.5)	89 (7.2)		8 (3.8)	74 (6.0)	21 (12.7)		39 (5.8)	60 (7.2)	
Unknown	41	8	33		12	26	3		10	21	
Type of costs due to MG: Transportation costs, n (%)				− 0.37				0.70			0.21
No answer	96 (6.1)	16 (4.3)	80 (6.6)		16 (7.9)	76 (6.3)	2 (1.2)		35 (5.3)	49 (6.0)	
No, health insurance covers transport	63 (4.0)	8 (2.1)	55 (4.5)		4 (2.0)	40 (3.3)	18 (10.8)		16 (2.4)	42 (5.2)	
No, no costs are arising	701 (44)	215 (57.6)	486 (40.1)		132 (65.0)	536 (44.5)	32 (19.3)		329 (49.7)	333 (41.0)	
Yes	726 (46)	134 (35.9)	592 (48.8)		51 (25.1)	552 (45.8)	114 (68.7)		282 (42.6)	388 (47.8)	
Unknown	74	15	59		18	52	3		24	39	
Costs of home remodelling due to MG, *yes* n (%)	199 (14)	20 (5.7)	179 (16.0)	− 0.34	23 (12.4)	132 (11.8)	41 (26.3)	0.37	72 (11.5)	107 (14.4)	0.09
Unknown	192	37	155		35	141	13		58	110	
Care costs for children due to MG, *yes* n (%)	26 (2.0)	2 (0.6)	24 (2.4)	− 0.14	3 (1.8)	15 (1.5)	8 (5.9)	0.23	18 (3.0)	5 (0.8)	0.16
Unknown	341	73	268		54	251	33		84	223	

^1^Comparing gMG and oMG.

^2^Comparing standard and intensified treatment.

^3^Comparing EOMG and LOMG.

Around 80.0% of study participants stated that they have costs arising from their MG, with more than half (54.0%) paying less than 100 euros per month. Higher levels of self-funded costs were particularly common in the group of patients with intensified treatment, who also reported transportation costs more frequently (no MG-related treatment 25.1%, standard treatment 45.8%, intensified treatment 68.7%). The effect size for the different treatment groups for self-funded costs was medium (SMD 0.58) and large for transportation costs (SMD 0.7), whereas effect sizes for these comparisons between oMG and gMG were medium and small for EOMG and LOMG. Structural changes to the home due to MG were necessary in around 14.0%, more frequently in patients with intensified treatment (26.3%) compared to no MG-related treatment (12.4%) or standard treatment (11.8%). Overall, 2.0% of study participants reported additional childcare costs due to the MG. Among the 123 patients with children 13 years of age or younger (data from our former publication (Stein et al. 2023), this proportion was 21.1%.

Of the 260 employed (non-retired) study participants of the claims dataset, 57.3% had at least one MG-related absence in the first year after index (Table 4), with the standard (68.8%) and intensified treatment (60.0%) groups particularly affected compared to the no MG-related treatment group (34.9%). There was a positive association between intensity of treatment and days of absence from work in days per patient year (no MG-related treatment 20.8 days, 95% CI 19.7–21.8; standard treatment 79.3 days, 95% CI 77.9–80.8; intensified treatment 168.1 days, 95% CI 162.0–174.4). Working LOMG patients were absent from work less often than EOMG patients (LOMG EOMG 1.0, 95% CI 0.8–1.2) absence from work per patient-year), but the duration in days of an absence was longer in LOMG (LOMG 76.1, 95% CI 74.7–77.6 vs. EOMG 55.2, 95% CI 53.8–56.6 days per patient year).

**Table 4:** MG related absence from work (AW) in the first year after index in the claims dataset in overall study population and different subgroups.

	All incident n = 775	No MG-related treatment n = 229	Standard treatment n = 447	Intensified treatment n = 99	EOMG n = 120	LOMG n = 655
Patients included in this analysis (= not retired population), n (%)	260 (33.6)	83 (36.2)	157 (35.1)	20 (20.2)	112 (93.3)	148 (22.6)
Patients with ≥ 1 AW, n (%)	149 (57.3)	29 (34.9)	108 (68.8)	12 (60.0)	63 (56.3)	86 (58.1)
AW/patient-year, n (95% CI)	0.89 (0.77–1.01)	0.38 (0.26–0.54)	1.1 (1.0–1.3)	1.1 (0.7–1.7)	1.0 (0.8–1.2)	0.79 (0.65–0.95)
AW in days/patient-year, n (95% CI)	67.1 (66.1–68.2)	20.8 (19.7–21.8)	79.3 (77.9–80.8)	168.1 (162.0–174.4)	55.2 (53.8–56.6)	76.1 (74.7–77.6)

#### Health care resource utilization

MG related doctor visits per year amounted to a mean of 5.2 (SD 6.9) in the DMG dataset (Table 5), while MG related outpatient visits per patient-year in the first year after index based on claims data were 4.9 (95% CI 4.7–5.0) (Table 6). In the DMG dataset, participants with intensified treatment had more MG-related doctor visits per year (10.2, SD 12.1) than participants with standard treatment (4.8, SD 5.7) and those without MG-related treatments (2.8, SD 5.5). Similarly, analysis of claims data depicted more outpatient visits per patient year in the first year after index for patients with standard and intensified treatment, compared with no MG-related treatment (no MG-related treatment 1.6, 95% CI 1.4–1.8; standard treatment 6.0, 95% CI 5.8–6.3; intensified treatment 7.0, 95% CI 6.5–7.6), which was also true when only assessing outpatient neurologist care (visits per patient year: no MG-related treatment 0.40, 95% CI 0.32–0.50; standard treatment 2.3, 95% CI 2.2–2.5; intensified treatment 2.4, 95% CI 2.1–2.8). Overall, at time of the survey, a mean of 0.9 (SD 3.5) hospital stays and 0.2 (SD 2.5) emergency room (ER) visits within the last 12 months were reported in the DMG dataset, whereas 0.8 (95% CI 0.73–0.87) hospitalizations per patient-year and 0.36 (95% CI 0.31–0.40) ER visits per patient-year after index were observed from claims data. The use of inpatient services was higher for patients with more intensive treatment in the DMG dataset: the high intensity treatment group had 3.1 hospital stays in the past 12 months (SD 4.2) versus 0.5 (SD 3.4) in participants with standard treatment and 0.4 (SD 1.1) in those without MG-related treatment (Table 5). More than one hospitalization in the first year after index was also more common with intensified therapy in the claims dataset (no MG-related treatment 13.1%; standard treatment 61.5%; intensified treatment 82.8%). Further, duration of inpatient stays in days per patient-year was considerably longer for patients with intensified treatment at 47.6 days (95% CI 46.1–49.1) than both other treatment groups (no MG-related treatment 1.7, 95% CI 1.6–1.9; standard treatment 7.3, 95% CI 7.0–7.6). Patients with intensified treatment had one or more ER visits in the first year after index in 67.0% of cases and one or more intensive care unit (ICU) visit in 40.4% of cases in the first year after index based on medical claims data. Notably, this was more common with treatment escalation (≥ 1 ER visit, no MG-related treatment 6.1% vs. standard treatment 28.2%; at least 1 ICU stay, no MG-related treatment 0.9% vs. standard treatment 1.3%). For the comparison of HCRU of the different treatment intensity groups in the DMG dataset effect sizes were medium. There were only minor differences in effect size in the utilization of MG-related health resources between the EOMG and LOMG groups in DMG data. In claims data, ICU stays were more common in LOMG than EOMG patients (EOMG 0.8%, LOMG 7.2%) though ICU stays per patient-year were still relatively low at 0.01 (95% CI 0.00–0.04) for EOMG and 0.10 for LOMG (95% CI 0.08–0.13). Comparing study participants with oMG and gMG in the DMG dataset, gMG patients had on average more MG-related doctor visits (oMG 3.9, SD 3.3 vs. gMG 5.6, SD 7.6) and hospital stays (oMG 0.4, SD 1.0 vs. gMG 1.0, SD 3.9) while ER visits (oMG 0.4, SD 5.0 vs gMG 0.2, SD 0.8) were similarly frequent though effect sizes were small.

**Table 5:** MG related health care resource utilization (HCRU) in the DMG dataset in overall study population and different subgroups.

MG related HCRU per patient	overall n = 1,660	oMG n = 388	gMG n = 1,272	SMD^1^	No MG-related treatment n = 221	Standard treatment n = 1,256	Intensified treatment n = 169	SMD^2^	EOMG n = 686	LOMG n = 851	SMD^3^
Doctor visits per year, mean (SD)	5.2 (6.9)	3.9 (3.3)	5.6 (7.6)	− 0.27	2.8 (5.5)	4.8 (5.7)	10.2 (12.1)	− 0.58	4.9 (7.2)	5.3 (6.7)	− 0.05
Median (IQR)	4.0 (2.0, 6.0)	4.0 (2.0, 4.0)	4.0 (2.0, 6.0)		2.0 (1.0, 4.0)	4.0 (2.0, 5.0)	6.0 (4.0, 12.0)		4.0 (2.0, 5.3)	4.0 (2.0, 6.0)	
Unknown	100	18	82		31	59	8		30	43	
Hospital stays during past 12 months, mean (SD)	0.9 (3.5)	0.4 (1.0)	1.0 (3.9)	− 0.22	0.4 (1.1)	0.5 (3.4)	3.1 (4.2)	− 0.69	0.8 (2.3)	0.9 (4.4)	− 0.01
Median (IQR)	0.0 (0.0, 1.0)	0.0 (0.0, 0.0)	0.0 (0.0, 1.0)		0.0 (0.0, 0.0)	0.0 (0.0, 0.0)	2.0 (1.0, 4.0)		0.0 (0.0, 1.0)	0.0 (0.0, 1.0)	
Unknown	418	112	306		61	335	21		133	232	
ER visits during past 12 months, mean (SD)	0.2 (2.5)	0.4 (5.0)	0.2 (0.8)	0.06	0.6 (6.6)	0.1 (0.7)	0.5 (0.9)	− 0.47	0.3 (3.7)	0.1 (0.4)	0.09
Median (IQR)	0.0 (0.0, 0.0)	0.0 (0.0, 0.0)	0.0 (0.0, 0.0)		0.0 (0.0, 0.0)	0.0 (0.0, 0.0)	0.0 (0.0, 1.0)		0.0 (0.0, 0.0)	0.0 (0.0, 0.0)	
Unknown	520	134	386		76	389	50		172	286	

^1^ Comparing gMG and oMG.

^2^ Comparing standard and intensified treatment.

^3^ Comparing EOMG and LOMG.

**Table 6:** MG related health care resource utilization (HCRU) in the first year after index in the claims dataset in overall study population and different subgroups.

MG related HCRU:	All incident n = 775	No MG-related treatment n = 229	Standard treatment n = 447	Intensified treatment n = 99	EOMG n = 120	LOMG n = 655
Totally observed patient years, n	688.6	197.3	409.3	82.1	110.4	578.2
Patients with ≥ 1 outpatient visit, n (%)	578 (74.6)	107 (46.7)	390 (87.3)	81 (81.8)	96 (80.0)	482 (73.6)
Outpatient visits/patient-year, n (95% CI)	4.9 (4.7 – 5.0)	1.6 (1.4–1.8)	6.0 (5.8–6.3)	7.0 (6.5–7.6)	4.6 (4.3–5.1)	4.9 (4.7–5.1)
Including:						
Patients with ≥ 1 GP visit, n (%)	375 (48.4)	44 (19.2)	267 (59.7)	64 (64.7)	69 (57.5)	306 (46.7)
GP visits/patient-year, n (95% CI)	1.9 (1.8 – 2.0)	0.6 (0.5–0.8)	2.2 (2.1–2.4)	3.1 (2.8–3.5)	2.0 (1.7–2.2)	1.9 (1.8 – 2.0)
Patients with ≥ 1 neurologist visit, n (%)	413 (53.3)	36 (15.7)	308 (68.9)	69 (69.7)	60 (50.0)	353 (53.9)
Neurologist visits/patient-year, n (95% CI)	1.8 (1.7–1.9)	0.40 (0.32–0.50)	2.3 (2.2–2.5)	2.4 (2.1–2.8)	1.7 (1.5–1.9)	1.8 (1.7–1.9)
Patients with ≥ 1 ER visit, n (%)	207 (26.7)	14 (6.1)	126 (28.2)	67 (67.7)	24 (20.0)	183 (27.9)
ER visits/patient-year, n (95% CI)	0.36 (0.31–0.40)	0.07 (0.04–0.12)	0.34 (0.28–0.40)	1.1 (0.9–1.4)	0.24 (0.16–0.34)	0.38 (0.33–0.43)
Patients with ≥ 1 hospitalization, n (%)	387 (49.9)	30 (13.1)	275 (61.5)	82 (82.8)	58 (48.3)	329 (50.2)
Hospital stays/patient-year, n (95% CI)	0.80 (0.73–0.87)	0.15 (0.10–0.21)	0.83 (0.74–0.92)	2.19 (1.89–2.53)	0.82 (0.67–1.01)	0.79 (0.72–0.87)
Duration of inpatient stays, days/patient-year (95% CI)	10.5 (10.3–10.8)	1.7 (1.6–1.9)	7.3 (7.0–7.6)	47.6 (46.1–49.1)	5.0 (4.6–5.4)	11.6 (11.3–11.8)
Patients with ≥ 1 ICU stay, n (%)	48 (6.2)	2 (0.9)	6 (1.3)	40 (40.4)	1 (0.8)	47 (7.2)
ICU stays/patient-year, n (95% CI)	0.09 (0.07–0.11)	0.01 (0.00–0.03)	0.01 (0.01–0.03)	0.62 (0.47–0.81)	0.01 (0.00–0.04)	0.10 (0.08–0.13)

More than half of study participants owned a severely disabled person´s pass (62.6%), which was more common with higher intensity of MG therapy. In contrast, most study participants had no care level (84%), with care level 2 being the most common (9%) when present (Supplement 4). Participants with intensified treatment had a higher level of care with no care level in 69.9% in this group compared with 86.8% in the group with no MG-related treatment and 85.6% in the group with standard treatment. Medical aids were requested by 19.3% of study participants. About one-fifth of requests for medical aids were rejected by the health insurance companies. In the gMG and intensified treatment groups, requests for medical remedies were more common than in the other MG subgroups.

#### MG-related costs

**Fig. 3:**
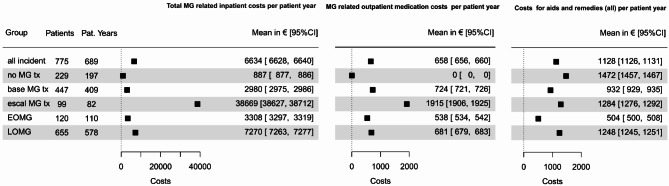
MG-related costs per patient year in the first year after index in the claims dataset in overall study population and different subgroups: MG tx = no MG-related treatment, base MG tx = standard treatment, escal MG tx = intensified treatment.

Overall costs per patient year resulting from inpatient stays (6634€, 95% CI 6628–6640€) were higher than outpatient medication costs (658€, 95% CI 656–660€) as well as costs for aids and remedies (1128€, 95% CI 1126–1131€) (claims data, Fig. 3). Mean inpatient costs per patient year were especially high in the group of patients with intensified treatment (38,669€, 95% CI 38,627–38,712€) compared with no MG-related treatment (887€, 95% CI 877–886€) and standard treatment (2980€, 95% CI 2975–2986€). Mean outpatient medication costs per patient year were also higher for patients with intensified treatment (1915€, 95% CI 1906–1925€) than no MG-related treatment (0€) and standard treatment (724€, 95% CI 721–726€). Costs of aids and remedies per patient year however did not show substantial differences between the 3 groups (no MG-related treatment 1472€, 95% CI 1457–1467€; standard treatment 932€, 95% CI 929–935€; intensified treatment 1284€, 95% CI 1276–1292€). All MG related costs per patient year were higher in patients with LOMG than in those with EOMG (inpatient costs 7270€, 95% CI 7263–7277€ vs. 3308€, 95% CI 3297–3319€; outpatient medication costs 681% CI €679–683€ vs. 538€, 95% CI 534–542€ costs for aids and remedies 1248€, 95% CI 1245–1251€ vs. 504€, 95% CI 500–508€).

## Discussion

The aim of this study was to provide a comprehensive understanding of the economic burden of Myasthenia Gravis (MG) from both a personal and societal perspective. This would not have been possible with a single dataset, as the available datasets focus exclusively on one perspective. By integrating a questionnaire-based approach for personal burden (n = 1660) and a claims dataset for healthcare costs (n = 775), we addressed both dimensions. In this study we demonstrate that individual as well as societal economic burden due to MG is high including more frequent physician visits, increased days of sickness with sick pay claims, more hospitalizations, and the use of expensive pharmacological therapies. Patients that receive intensified treatment are particularly affected by this and cost of hospitalisations in this group is a major cost driving factor.

Individual economic burden is evident in income losses reported by 30% of patients. More than half of working MG patients experienced restrictions on employment. Income decreases as well as unemployment were previously reported in a Japanese cohort, where effects were even stronger^16^. Income loss was also reported as a major concern for MG patients in a small cohort in the U.S.^17^. Income losses of approximately 30% have also been reported in other immunological neurological diseases such as multiple sclerosis (MS) and chronic inflammatory demyelinating polyneuropathy (CIDP), but usually only after disease progression^18,19^. In contrast, in MG, substantial income losses can occur much earlier and may cause occupational setbacks even in the initial stages of the disease. The fluctuating nature of MG often complicates the assessment of eligibility for severely disabled person’s pass and care levels. While there is no literature directly addressing this issue, our clinical experience suggests that the absence of objective measurement systems to adequately capture MG-related impairments makes the process vulnerable to under- and overestimations. Consequently, patients frequently face systemic disadvantages, as evidenced by our finding that 84% of MG patients had no care level assigned, and most of those classified only received care level 2. Clinical experience further indicates that appeals are often necessary to secure appropriate classifications. In our study, 80% of participants reported additional monthly self-covered costs due to MG and almost half stated transportation costs that were not covered by health insurance. For a subset of patients, home-remodelling became necessary which has been reported before for rheumatoid arthritis as a relevant cost-point and likely represents patients with restricted mobility^20^. Self-covered costs by patients vary widely by country and are, in general, lower in developed countries than in developing countries, where patients may have to cover more expenses on their own^21,22^. For Germany, one previous study estimated annual co-payments for MG patients in 2009 at a mean of 280 €, which may be largely reflected in our cohort with 20% of patients reporting no self-covered costs and 54% reporting less than 100€ monthly self-covered expenses due to MG^5^. Another study from 2010, estimated monthly self-covered costs due to MG at a median of 50 Euros^6^. However, a direct comparison is not possible because analyses were performed at different points in time and only monthly or annual self-covered costs are given. In addition to the self-covered costs that directly affect the patient, carers may also face independent economic burdens due to care work leading to elevated family economic burden^23^. Interestingly, in our cohort 25% reported income not to be sufficient for the same standard of living as before MG. The majority though (75%) may have well-functioning support from the social security system in the event of absence from work at working-age. However, in the group of patients with EOMG, likely representing working patients in particular, fear of poverty in old age was prevalent. The economic burden of MG on working patients has personal but also societal implications resulting from inabilities to work. Claims data revealed long periods of absence from work (67.1 days per patient year) for MG patients in the first year after index. In comparison, in the general German population between 2014 and 2019 the average number of days of absence per employee per year was between 9.5 and 10.9^24^. Risk of long-term sickness absences has been shown to be elevated in chronic diseases in general and for neurological diseases (excluding stroke, paraplegia and hemiplegia) in particular absences from work were high in the first year after diagnosis and reduced in a moderate to small way afterwards indicating prolonged risk of work limitations with some possibility of improvement^25^. This is in line with a recent Swedish cohort study, that indicated absences from work in MG to be particularly high in the first year after diagnosis^26^.

Our results reveal a strong association of treatment escalation with economic burden. Patients with intensified treatment were more severely affected as indicated by absences from work in days per patient-year more than twice as high as patients with standard treatment and more than eight times higher than patients with no MG-related treatment. The more intensive the medical treatment for MG, the more likely people were to report a loss of income due to MG, and the fewer people reported that their income was sufficient to maintain the same standard of living as before MG. Only 6.6% of patients with intensified treatment reported no self-covered costs. Societal costs in health care utilization were highest for patients with intensified treatment. Although hospitalization rates were observed to be declining between 2017 and 2020 with a mean overall hospitalization rate of 11.5% in another study of German claims data, costs for hospitalizations were high especially in the group of patients with intensified treatment which is in line with a previous study that identified hospitalizations as major cost-driving factors in MG^21,27^. Similarly, in a previous study comparing costs arising from three different rare neurological diseases (MG, amyotrophic lateral sclerosis, facioscapulohumeral muscular dystrophy) disease severity was identified as a major factor of socioeconomic burden in MG and further hospitalization costs were particularly high in MG compared to both other diseases^5^. Compared to inpatient costs arising in the general population costs for inpatient stays in MG accounted for more than half of the mean total monthly cost difference in a recent US study^28^. The higher frequency of hospitalization and ICU visits that was observed in our study for patients with intensified treatment also matches observations of higher MG related costs due to exacerbations^29,30^. It is important to note that patients receiving intensified treatment are likely those with highly active disease, and it has been shown that early escalation of therapy, may lead to faster therapy de-escalation overall^31,32^. Fortunately, the treatment landscape for antibody-positive gMG has expanded in recent years and the data on intensified therapy no longer reflect all current treatment options. Our survey was conducted in 2019, so Rituximab was categorized under ‘intensified treatment’ due to its typical use in more active or therapy-refractory cases, as reflected in clinical practice and current German guidelines. Its role as a cost-effective alternative in specific cases highlights the need for further economic evaluations, particularly as newer therapies become more widely implemented. It is likely that these novel therapies will increase costs tremendously as indicated in one recent Chinese study on cost-effectiveness of eculizumab and efgartigimod treatment of MG where both exceeded common cost-effectiveness thresholds^33^ also evidenced by a meta-analysis indicating that the costs to achieve an improvement of more than three points in the MG-ADL (MG Activities of Daily Living score) range from approximately 800,000 USD for efgartigimod to over 2,500,000 USD for ravulizumab^34^. Additionally, there will be costs and burden to patients and caregivers resulting from e.g. transportation to specialized centers if infusions have to be administered in a hospital or at a specialized doctor’s office.

This study has several limitations. First, though we tried to harmonise both data sources and definitions of subgroups a direct integration of data from one source into the other was not possible. The DMG dataset was derived from questionnaires sent back anonymously therefore some data may have been influenced through recall bias or limited medical knowledge; the DMG population may not fully represent the average German MG patient, as it is slightly older and potentially includes more severely affected or highly motivated individuals. Regarding age though, both mean age (65.3 years in DMG dataset and 66.9 in claims dataset) are similar. Limitations such as selection bias, recall bias, and the lack of clinical validation due to anonymous questionnaires may have influenced the results, though efforts were made to minimize these effects by offering a long response time^7^. Claims data of incident patients allows isolated insight into the early disease course. In MG disease activity is known to be particularly high in the first years which might have skewed data towards more intensified therapies. Furthermore, some of the prescribed drugs could have been prescribed for other indications (e.g., other autoimmune diseases).

## Conclusion

In this study, utilizing two distinct datasets—one sourced from patients’ self-reports and the other from insurance records—we have demonstrated that the burden of Myasthenia Gravis (MG) encompasses an economic dimension. Specifically, persons affected by MG experience limitations in occupational engagement and income, while society faces significant financial burdens, particularly in cases of diseased young, working, individuals and severe disease progression. As expensive novel therapeutic options emerge, investigations into this domain are poised to become increasingly imperative in the coming years. Further studies would not only facilitate better resource allocation within healthcare systems but also inform policy measures aimed at alleviating the financial strain on both individuals and societies.

## Electronic supplementary material

Below is the link to the electronic supplementary material.

Supplementary Material 1

## Data Availability

The datasets used and/or analysed during the current study available from the corresponding author on reasonable request.

## Abbreviations

ATC: Anatomical therapeutic chemical classification system
CI: Confidence interval
CIDP: Chronic inflammatory demyelinating polyneuropathy
DMG: Deutsche Myasthenie Gesellschaft (German Myasthenia Society)
EOMG: Early onset Myasthenia gravis
EUR: Euro
ER: Emergency room
gMG: Generalized Myasthenia gravis
GP: General practitioner
HCRU: Health care resource utilization
ICU: Intensive care unit
LOMG: Late onset Myasthenia gravis
MG: Myasthenia gravis
MG-ADL: MG Activities of Daily Living score
MS: Multiple sclerosis
oMG: Ocular Myasthenia gravis
OPS: Operationen- und Prozedurenschlüssel (Operation and Procedure Classification System)
SD: Standard deviation
SMD: Standardized mean difference
USD: United States of America Dollar

## References

[1] Huijbers, M. G., Marx, A., Plomp, J. J., Le Panse, R. & Phillips, W. D. Advances in the understanding of disease mechanisms of autoimmune neuromuscular junction disorders. *Lancet Neurol.* **21**, 163–175 (2022). 35065039 10.1016/S1474-4422(21)00357-4

[2] Nelke, C. et al. Independent risk factors for Myasthenic crisis and disease exacerbation in a retrospective cohort of myasthenia gravis patients. *J. Neuroinflammation* **19**, 89 (2022). 35413850 10.1186/s12974-022-02448-4 PMC9005160

[3] Schneider-Gold, C., Hagenacker, T., Melzer, N. & Ruck, T. Understanding the burden of refractory myasthenia gravis. *Ther. Adv. Neurol. Disord.* **12**, 1756286419832242 (2019). 30854027 10.1177/1756286419832242 PMC6399761

[4] Iorio, R. Myasthenia gravis: the changing treatment landscape in the era of molecular therapies. *Nat. Rev. Neurol.* **20**, 84–98 (2024). 38191918 10.1038/s41582-023-00916-w

[5] Schepelmann, K. et al. Socioeconomic burden of amyotrophic lateral sclerosis, myasthenia gravis and facioscapulohumeral muscular dystrophy. *J. Neurol.* **257**, 15–23 (2010). 19629566 10.1007/s00415-009-5256-6

[6] Twork, S., Wiesmeth, S., Klewer, J., Pöhlau, D. & Kugler, J. Quality of life and life circumstances in German myasthenia gravis patients. *Health Qual. Life Outcomes* **8**, 129 (2010). 21070628 10.1186/1477-7525-8-129 PMC2994799

[7] Lehnerer, S. et al. Burden of disease in myasthenia gravis: Taking the patient’s perspective. *J. Neurol.* **269**, 3050–3063 (2022). 34800167 10.1007/s00415-021-10891-1 PMC9120127

[8] Mevius, A. et al. Epidemiology and treatment of myasthenia gravis: A retrospective study using a large insurance claims dataset in Germany. *Neuromuscul. Disord.* **33**, 324–333 (2023). 36921445 10.1016/j.nmd.2023.02.002

[9] Cuschieri, S. The STROBE guidelines. *Saudi J. Anaesth.* **13**, S31–S34 (2019). 30930717 10.4103/sja.SJA_543_18 PMC6398292

[10] TeleForm. Get the software safely and easily. *Softw. Inf.* (2024). <https://teleform.software.informer.com/10.5/>

[11] Burns, T. M., Conaway, M. R., Cutter, G. R., Sanders, D. B., Muscle Study Group. Less is more, or almost as much: A 15-item quality-of-life instrument for myasthenia gravis. *Muscle Nerve* **38**, 957–963 (2008). 18642357 10.1002/mus.21053

[12] Wolfe, G. I. et al. Myasthenia gravis activities of daily living profile. *Neurology* **52**, 1487–1489 (1999). 10227640 10.1212/wnl.52.7.1487

[13] IBM Corp. Released. *IBM SPSS Statistics for Windows, Version 25.0* (IBM Corp, 2017).

[14] RCore Team. _R: A Language and Environment for Statistical Computing_. *R Found. Stat. Comput.* https://www.r-project.org/

[15] Yoshida, K. & Bartel, A. tableone: Create ‘Table 1’ to Describe Baseline Characteristics with or without Propensity Score Weights. 0.13.2. 10.32614/CRAN.package.tableone (2014).

[16] Nagane, Y. et al. Social disadvantages associated with myasthenia gravis and its treatment: A multicentre cross-sectional study. *BMJ Open* **7**, e013278 (2017). 28235967 10.1136/bmjopen-2016-013278 PMC5337722

[17] Hughes, T. et al. The economic burden of individuals living with generalized myasthenia gravis and facing social determinants of health challenges. *Front. Public Health* **11**, 1247931 (2023). 37766748 10.3389/fpubh.2023.1247931 PMC10520715

[18] Leray, E. et al. Impact of multiple sclerosis on employment and income: Insights from a random sample representative of private sector employees in France using longitudinal administrative data. *Rev. Neurol. (Paris)* **180**, 754–765 (2024). 38582662 10.1016/j.neurol.2024.02.389

[19] Mengel, D. et al. Costs of illness in chronic inflammatory demyelinating polyneuropathy in Germany. *Muscle Nerve* **58**, 681–687 (2018). 30073683 10.1002/mus.26315

[20] Gabriel, S. E., Crowson, C. S., Campion, M. E. & O’Fallon, W. M. Indirect and nonmedical costs among people with rheumatoid arthritis and osteoarthritis compared with nonarthritic controls. *J. Rheumatol.* **24**, 43–48 (1997). 9002009

[21] Ignatova, V. et al. Socio-economic burden of Myasthenia gravis: A cost-of-illness study in Bulgaria. *Front. Public Health* **10**, 822909 (2022). 35309194 10.3389/fpubh.2022.822909 PMC8927679

[22] Sonkar, K. K., Bhoi, S. K., Dubey, D., Kalita, J. & Misra, U. K. Direct and indirect cost of myasthenia gravis: A prospective study from a tertiary care teaching hospital in India. *J. Clin. Neurosci. Off. J. Neurosurg. Soc. Australas.* **38**, 114–117 (2017). 10.1016/j.jocn.2016.11.003 27887977

[23] Meng, D.-D. et al. Factors associated with the disease family burden of caregivers of myasthenia gravis patients in northwestern China: A cross-sectional study. *J. Clin. Neurosci. Off. J. Neurosurg. Soc. Australas.* **119**, 70–75 (2024). 10.1016/j.jocn.2023.11.024 37988975

[24] Institut für Arbeitsmarkt- und Berufsforschung. Krankenstand. *Stat. Bundesamt* at <https://www.destatis.de/DE/Themen/Arbeit/Arbeitsmarkt/Qualitaet-Arbeit/Dimension-2/krankenstand.html>

[25] Nexo, M. A. et al. Long-term sickness absence of 32 chronic conditions: A Danish register-based longitudinal study with up to 17 years of follow-up. *BMJ Open* **8**, e020874 (2018). 29961016 10.1136/bmjopen-2017-020874 PMC6042549

[26] Cai, Q. et al. Long-term healthcare resource utilization and costs among patients with myasthenia gravis: A Swedish nationwide population-based study. *Neuroepidemiology*10.1159/000538640 (2024). 38631321 10.1159/000538640 PMC11633887

[27] Wartmann, H. et al. Incidence, prevalence, hospitalization rates, and treatment patterns in myasthenia gravis: A 10-year real-world data analysis of German claims data. *Neuroepidemiology* **57**, 121–128 (2023). 36807212 10.1159/000529583 PMC10129022

[28] Zhdanava, M. et al. Economic burden of generalized myasthenia gravis (MG) in the United States and the impact of common comorbidities and acute MG-events. *Curr. Med. Res. Opin.* **40**, 1145–1153 (2024). 38745448 10.1080/03007995.2024.2353381

[29] Antonini, G. et al. Real world study on prevalence, treatment and economic burden of myasthenia gravis in Italy. *Heliyon* **9**, e16367 (2023). 37274644 10.1016/j.heliyon.2023.e16367 PMC10238888

[30] Ting, A. et al. A real-world analysis of factors associated with high healthcare resource utilization and costs in patients with myasthenia gravis receiving second-line treatment. *J. Neurol. Sci.* **445**, 120531 (2023). 36634582 10.1016/j.jns.2022.120531

[31] Piehl, F. et al. Efficacy and safety of rituximab for new-onset generalized myasthenia gravis: The RINOMAX randomized clinical trial. *JAMA Neurol.* **79**, 1105–1112 (2022). 36121672 10.1001/jamaneurol.2022.2887 PMC9486640

[32] Utsugisawa, K. et al. Early fast-acting treatment strategy against generalized myasthenia gravis. *Muscle Nerve* **55**, 794–801 (2017). 27603432 10.1002/mus.25397 PMC5484288

[33] Lien, P.-W. et al. Cost-effectiveness of eculizumab and efgartigimod for the treatment of anti-acetylcholine receptor antibody-positive generalized myasthenia gravis. *J. Manag. Care Spec. Pharm.* **30**, 517–527 (2024). 38824625 10.18553/jmcp.2024.30.6.517 PMC11144987

[34] Smith, A. G. et al. Risk-benefit analysis of novel treatments for patients with generalized myasthenia gravis. *Adv. Ther.* **41**, 4628–4647 (2024). 39470879 10.1007/s12325-024-03014-5 PMC11550228

