# Supplementary material for: The economic burden of Myasthenia gravis from the patient´s perspective and reflected in German claims data
Source: Sci Rep. 2025 Feb 25;15:6687. doi: 10.1038/s41598-025-91372-7 (PMC11850840; doi:10.1038/s41598-025-91372-7)
Supplement: Supplementary file 1 — Supplementary Material 1 [file 41598_2025_91372_MOESM1_ESM.docx]

## Supplementary Materials

Supplement 1 **Identification codes** for standard of care-MG treatment

| **Treatment** | **Identification (ATC/procedure code)** |
| --- | --- |
| Oral corticosteroids | ATC H02AB- |
| Pyridostigmine | ATC N07AA02 |
| Azathioprine | ATC L04AX01 |
| Mycophenolate mofetil | ATC L04AA06 |
| Cyclosporine | ATC L04AD01 |
| Rituximab | ATC L01XC02; procedure code (OPS) 6-001.h |
| Eculizumab | ATC L04AA25; procedure code (OPS) 6-003.h |
| Intravenous immunoglobulin (IVIg) | ATC J06BA02; procedure code (OPS) 8-810.w |
| Plasma exchange (PLEX) | procedure code (OPS) 8-820 |
| Immunoadsorption (IA) | procedure code (OPS) 8-821 |
| Thymectomy | procedure code (OPS) 5-077 |

Supplement 2 **MG-ADL score** (Mean, SD) in the DMG dataset in overall study population and different subgroups.
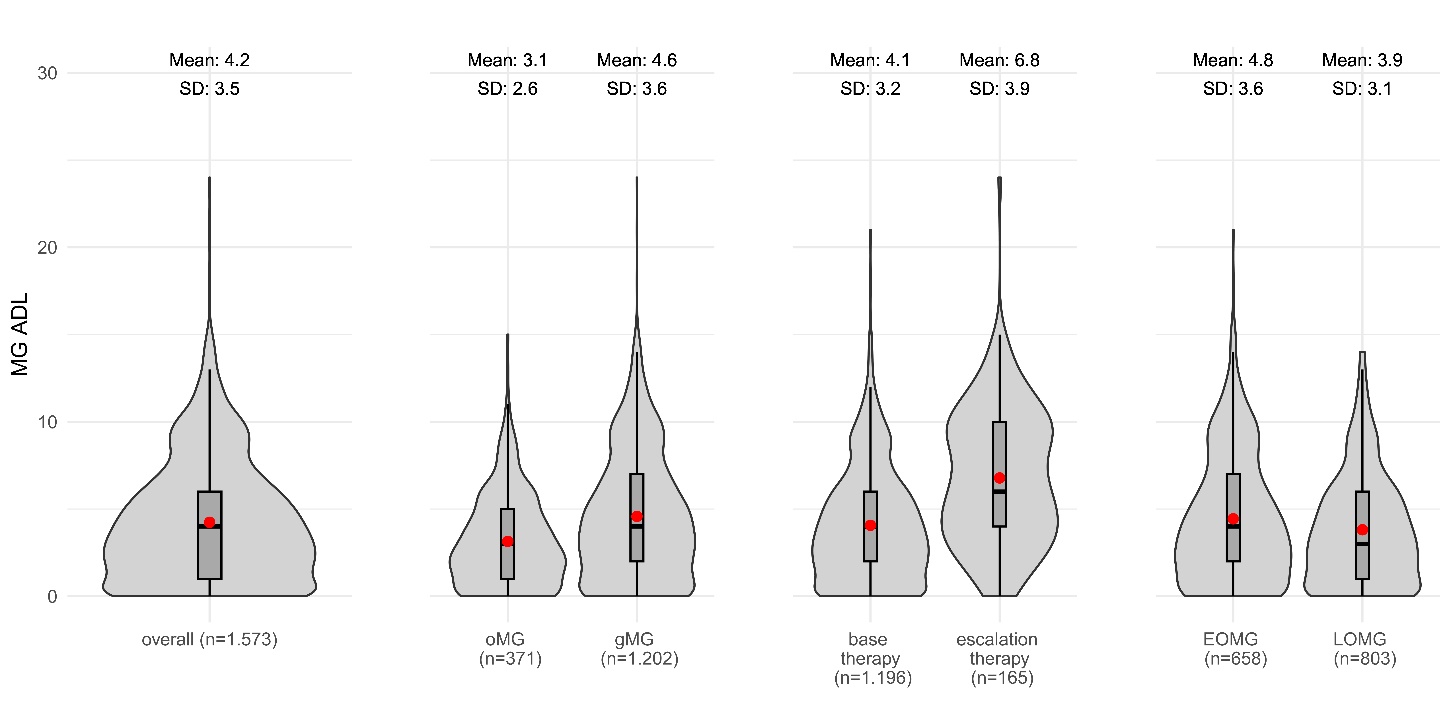


Supplement 3 **MG-QoL15 score** (Mean, SD) in the DMG dataset in overall study population and different subgroups.
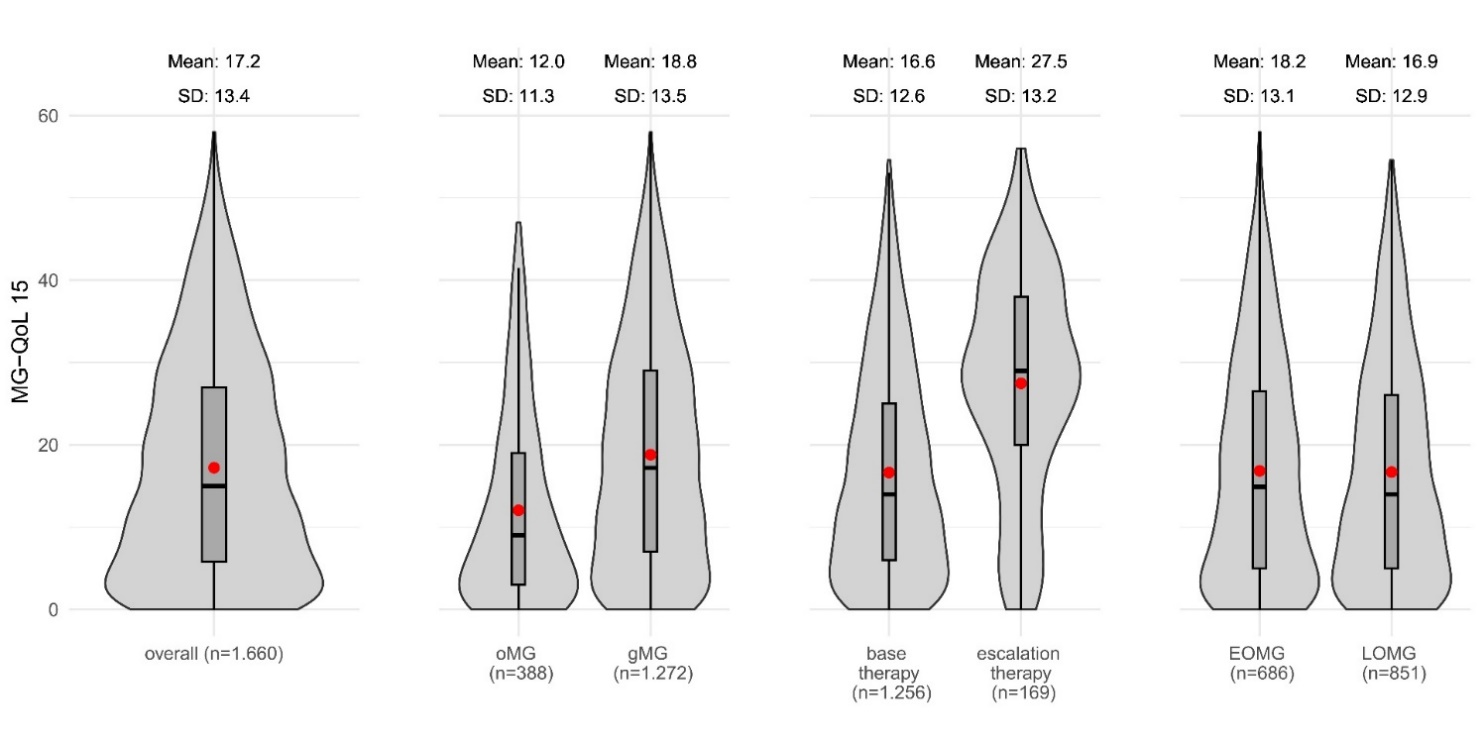


Supplement 4 **Type of residency, care dependency level and medical aids and remedies** of the DMG dataset in overall study population and different subgroups

|  | **overall**  n = 1,660 | **oMG**  n = 388 | **gMG**  n = 1,272 | **SMD^1^** | **no MG-related treatment**  n = 221 | **standard treatment**  n = 1,256 | **intensified treatment**  n = 169 | **SMD^2^** | **EOMG**  n = 686 | **LOMG**  n = 851 | **SMD^3^** |
| --- | --- | --- | --- | --- | --- | --- | --- | --- | --- | --- | --- |
| **Care level,** n% |  |  |  | -0.27 |  |  |  | 0.45 |  |  | 0.18 |
| No care dependency level | 1,370 (84.0) | 345 (90.3) | 1,025 (82.1) |  | 190 (86.8) | 1,055 (85.6) | 116 (69.9) |  | 595 (87.9) | 688 (82.4) |  |
| Care dependency level 1 | 33 (2.0) | 8 (2.1) | 25 (2.0) |  | 5 (2.3) | 26 (2.1) | 2 (1.2) |  | 7 (1.0) | 20 (2.4) |  |
| Care dependency level 2 | 146 (9.0) | 16 (4.2) | 130 (10.4) |  | 14 (6.4) | 96 (7.8) | 33 (19.9) |  | 49 (7.2) | 80 (9.6) |  |
| Care dependency level 3 | 52 (3.2) | 8 (2.1) | 44 (3.5) |  | 8 (3.7) | 34 (2.8) | 10 (6.0) |  | 17 (2.5) | 30 (3.6) |  |
| Care dependency level 4 | 6 (0.4) | 1 (0.3) | 5 (0.4) |  | 1 (0.5) | 4 (0.3) | 0 (0.0) |  | 1 (0.1) | 3 (0.4) |  |
| Care dependency level 5 | 3 (0.2) | 1 (0.3) | 2 (0.2) |  | 0 (0) | 3 (0.2) | 0 (0.0) |  | 0 (0.0) | 3 (0.4) |  |
| No, but a request has been made | 21 (1.3) | 3 (0.8) | 18 (1.4) |  | 1 (0.5) | 15 (1.2) | 5 (3.0) |  | 8 (1.2) | 11 (1.3) |  |
| Unknown | 29 | 6 | 23 |  | 2 | 23 | 3 |  | 9 | 16 |  |
| **Severely disabled person’s pass granted,** n% |  |  |  | -0.39 |  |  |  | 0.31 |  |  | 0.22 |
| No | 544 (33.1) | 181 (47.0) | 363 (28.8) |  | 106 (48.6) | 403 (32.3) | 32 (19.2) |  | 192 (28.2) | 321 (3.18) |  |
| No, but requested | 72 (4.4) | 10 (2.6) | 62 (4.9) |  | 2 (0.9) | 59 (4.7) | 11 (6.6) |  | 28 (4.1) | 39 (4.6) |  |
| Yes | 1,029 (62.6) | 194 (50.4) | 835 (66.3) |  | 110 (50.5) | 784 (62.9) | 124 (74.3) |  | 462 (67.7) | 483 (57.3) |  |
| Unknown | 15 | 3 | 12 |  | 3 | 10 | 2 |  | 4 | 8 |  |
| **Mean level of disability,** mean (SD) | 64.3 (19.8) | 59.1 (18.2) | 65.6 (20.0) | -0.34 | 62.5 (20.7) | 63.5 (19.7) | 70.6 (18.9) | -0.37 | 61.8 (19.9) | 66.3 (19.3) | -0.23 |
| Unknown | 675 | 203 | 472 |  | 116 | 503 | 53 |  | 234 | 398 |  |
| **Applied for medical aids,** *yes* n (%) | 314 (19.3) | 42 (11.0) | 272 (21.8) | -0.30 | 26 (12.2) | 219 (17.7) | 62 (37.1) | -0.44 | 133 (19.6) | 144 (17.3) | 0.06 |
| Unknown | 32 | 5 | 27 |  | 8 | 22 | 2 |  | 7 | 17 |  |
| **If yes: Medical aids rejected,** *yes* n (%) | 62 (20.4) | 7 (17.9) | 55 (20.8) | -0.07 | 3 (11.5) | 46 (21.9) | 12 (19.7) | 0.06 | 34 (25.8) | 22 (16.2) | 0.24 |
| N.a./Unknown | 1,356 | 349 | 1,007 |  | 195 | 1,046 | 108 |  | 554 | 715 |  |
| **Applied for medical remedies,** *yes* n (%) | 601 (37.4) | 79 (20.8) | 522 (42.5) | -0.48 | 50 (24.2) | 441 (36.1) | 100 (59.9) | 0.49 | 266 (39.5) | 282 (34.3) | 0.11 |
| Unknown | 53 | 9 | 44 |  | 14 | 35 | 2 |  | 13 | 30 |  |
| **If yes: Medical remedies rejected,** *yes* n (%) | 95 (15.8) | 13 (16.9) | 82 (15.6) | 0.04 | 6 (12.5) | 73 (16.3) | 15 (15.2) | 0.03 | 44 (16.5) | 38 (13.4) | 0.09 |
| N.a./Unknown | 1,057 | 311 | 746 |  | 173 | 809 | 70 |  | 419 | 568 |  |

Abbreviations. DMG=Deutsche Myasthenie Gesellschaft (German myasthenia gravis association), MG=Myasthenia gravis, SD=Standard Deviation, SMD=Standardized mean difference, gMG=generalized Myasthenia gravis, oMG=ocular Myasthenia gravis, EOMG=Early onset myasthenia gravis, LOMG=Late onset myasthenia gravis
